# Ecology and evolution of migration in the freshwater eels of the genus *Anguilla* Schrank, 1798

**DOI:** 10.1016/j.heliyon.2020.e05176

**Published:** 2020-10-06

**Authors:** Takaomi Arai

**Affiliations:** a Universiti Brunei Darussalam, Jalan Tungku Link, Gadong, BE 1410, Brunei Darussalam; b Universitas Airlangga, Surabaya, 60113, Indonesia

**Keywords:** *Anguilla*, Catadromy, Continental migration, Diversity, Oceanic migration, Spawning, Agricultural science, Animal science, Environmental science, Ecology, Biological sciences, Zoology

## Abstract

Scientists have long sought to uncover the secrets of the migration of anguillid eels, genus *Anguilla*. As catadromous fishes, anguillid eels spend most of their lives in freshwater until they return to their spawning grounds in the tropics, although part of the population never enters freshwater and instead resides in brackish and marine areas close to coastlines. Molecular phylogenetic research suggests that anguillid eels originated from deep-ocean midwater marine anguilliform species and that tropical eels originating from the Indo-Pacific region are the most basal species of anguillid eels. Anguillid eels left the tropical ocean to colonize temperate areas. The yearly spawning of tropical species and constant larval growth throughout the year extend to periods of recruitment in continental habitats to last all year for tropical eels. Tropical eels such as *A. celebesensis* and *A. borneensis* have relatively short migrations periods of less than 100 km to their spawning grounds. Conversely, the temperate European eel *A. anguilla* travels the longest distances and migrates more than 5000 km across the Atlantic Ocean to spawn in the Sargasso Sea. The ancestral state of migration in the genus *Anguilla* may have been local, short-scale and nonseasonal spawning migration throughout the year as defined in tropical eels. With the expansion of dispersion of global oceanic migration across the world, migration scales can gradually change. Temperate anguillid eels migrate thousands of kilometres from spawning areas to coastal and inland water habitats while retaining spawning areas in tropical areas, accompanied by seasonal downstream and spawning migrations with consequences for seasonal recruitment. Recent advances and the availability of electronic tags such as pop-up satellite archival tag could reconstruct the entire spawning migration from continental growth habitats to spawning sites with detailed migration behaviours and routes. Migration ecology and mechanisms throughout the life of anguillid eels have gradually been revealed in recent decades.

## Teaser

*Anguilla*, catadromy, continental migration, diversity, oceanic migration, spawning, Agricultural Science, Animal Science, Environmental Science, Ecology, Biological Sciencesy, Zoology.

## 1 Introduction

**Figure 1:**
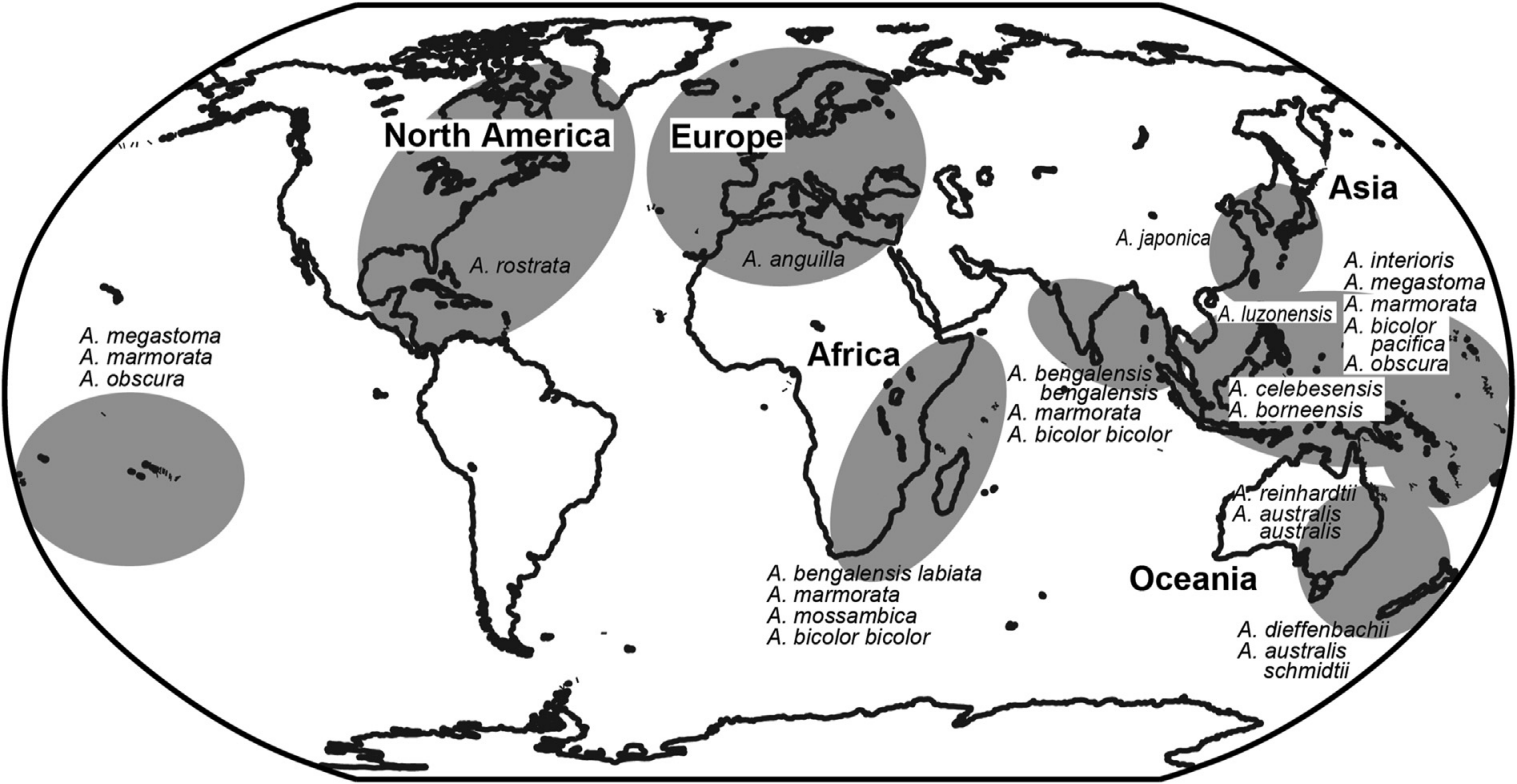
Geographical distribution of the 19 species and subspecies of genus *Anguilla*.

Anguillid eels, genus *Anguilla* Schrank, 1798, represent one of the most unique eel groups of the Anguilliformes, consisting of 20 families, 147 genera and 820 species (Nelson, 2006; Johnson et al., 2011). Anguillid eels are the only genus in which all species lead catadromous life patterns of migrating between coastal and inland water growth habitats and offshore spawning areas (McDowall, 1988). They spawn in tropical oceans and their transparent larvae, leptocephali, passively drift through currents from offshore spawning areas to their coastal and inland water habitats. A recent study found that the majority of leptocephali of the Atlantic eels *A. anguilla* and *A. rostrata* remain trapped and possibly dies in the retention area of the Gulf Stream system (Westerberg et al., 2018). The results suggest that the spawning success would be highly sensitive to oceanographic and climatic factors that alter the dispersion of leptocephali out from the retention area (Westerberg et al., 2018). This form of larval transport can result in large-scale dispersal, which can be affected by shifts in oceanic currents and can result in the transport of larvae outside of their typical species range. Anguillid eels are broadly found in most tropical and subtropical waters across the world (Figure 1). The freshwater distribution of the temperate anguillids appears to be closely involved in the subtropical gyre circulation, with most anguillids being found along western regions of the Atlantic, Pacific and Indian Oceans with some exceptions (Ege, 1939, Figure 1). Anguillid eels are not distributed over eastern South America, although Brazil Current flows southwestward along the coast. The Atlantic eels, *A. anguilla* and *A. rostrata*, are spatially divided from other anguillids in the Indo-Pacific region. Such a unique biogeography and species distribution have attracted considerable attention recently, and many studies on ecology, molecular phylogenetics and population structure have been examined.

Nineteen species and subspecies of the genus *Anguilla* have been reported worldwide (Ege, 1939; Castle and Williamson, 1974; Watanabe et al., 2004, 2009, 2013, 2014a, b; Arai, 2016, Figure 1). Anguillid eels can be divided into “tropical eels” and “temperate eels” based on their geographical continental habitats (Tesch, 1977). Among the 19 species/subspecies, the spawning migration patterns of *A. anguilla* and *A. rostrata* in the Atlantic Ocean and *A. japonica* in the northern Pacific Ocean have been studied due to their long distances and commercial importance of the species. *A. anguilla* and *A. rostrata* migrate more than 2000–8000 km for spawning migration in the Sargasso Sea (Schmidt, 1922; Miller et al., 2019). The spawning area of *A. japonica* has been consequently discovered in the northwestern Pacific Ocean, and the eel migrates approximately 2000–4000 km to its growth habitat (Tsukamoto, 1992). Interestingly, the spawning area of a tropical eel *A. marmorata* has been found to overlap with that of a temperate eel *A. japonica* (Tsukamoto et al., 2011). Tropical anguillid eels, *A. celebesensis* and *A. borneensis*, distributed in tropical Southeast Asia regions, exhibit very short and local migrations less than 100 km from to their continental habitats (Aoyama et al., 2003, 2018; Arai, 2014a). Anguillid eels display various scales in oceanic migrations, from less than one hundred to thousands of kilometres. The long migration paths of temperate species and even the short migration paths of tropical eels are notable because of their scale and the excellent ability to find the birth location using an unrevealed combination of sensory cues (McCleave and Kleckner, 1985).

Various studies present different results regarding the most likely dispersal paths and the most basal eel species (Bastrop et al., 2000; Aoyama et al., 2001; Lin et al., 2001; Minegishi et al., 2005; Tseng et al., 2009; Tseng, 2016). Anguillid eels are believed to originate from the Indo-Pacific region. Higher marine biodiversity is found to occur in the Indo-Malay-Philippines Archipelago within the Indo-Pacific region (Carpenter and Springer, 2005). Indeed, two-thirds of anguillid eel species are also distributed in the Indo-Pacific Ocean. The reason for the high biodiversity discovered in the region is considered to be due to the area's frequent vicariant and island integration (Carpenter and Springer, 2005).

Phylogenetic relationships with integration of current distributions can provide valid information for the movement, dispersion and migration of species. Based on molecular phylogenetic studies of anguillid eels, *A. borneensis* and *A. mossambica* are believed to be the most plausible ancestral species (Aoyama et al., 2001; Lin et al., 2001; Minegishi et al., 2005; Teng et al., 2009; Tseng, 2016; Zan et al., 2020), which distribute in the Indo-Pacific Ocean. The Indo-Pacific Ocean is considered as an origin of speciation in anguillid eels, and tropical ones are certainly more closely connected to the basal form than temperate ones. However, compared to temperate anguillid eels, information regarding the basic biology and ecology of tropical anguillid eels is only at a rudimentary level. Therefore, studying the biology and ecology of tropical anguillid eels together with temperate anguillid eels could provide a better understanding of the ancestral migration in anguillid eels and how temperate eels have been acquired the capacity for long-distance migration.

The life cycle of anguillid eels involves five developmental stages: leptocephalus (larva), glass eel (transparent juvenile stage), elver (pigmented juvenile stage), yellow eel (immature adult) and silver eel (mature adult) (Bertin, 1956; Tesch, 1977; Cresci, 2020). Metamorphosis from leptocephalus to glass eel is one of the most significant life events. The initiation of metamorphosis and the leptocephalus period seem to constitute an essential biological role to determine the continental distribution (Tsukamoto, 1990; Arai et al., 2001). The lengthy duration of the larval stage might lead to global dispersion and their consequent speciation. The durations of leptocephalus stages span from several months to approximately two years (e.g., Tsukamoto, 1990; Lecomte-Finiger, 1992; Cheng and Tzeng, 1996; Arai et al., 1999a, 2001; 2003a; Wang and Tzeng, 2000; Marui et al., 2001; Robinet et al., 2003a; Robinet et al., 2008; Réveillac et al., 2008, 2009; Bonhommeau et al., 2010; Han et al., 2016a, 2019; Hewavitharane et al., 2020). Many marine organisms have a planktonic larval stage during their early life and larvae are passively transported by oceanic currents. This larval period generally suffers high mortality and subsequently has an influence on recruitment success (Lasker, 1975; Cushing, 1990; Durant et al., 2007). Leptocephali develop larger sizes and undergo a longer larval period than other fish larvae (Moran, 1994; Arai et al., 2001; Miller, 2009). The global dispersion was established due to the longer leptocephalus stage and passive transportation by means of oceanic currents and wind and subsequently extended their distribution nearly worldwide in the genus *Anguilla* (Figure 1).

After metamorphosis into glass eels, juveniles leave oceanic currents and then recruit to coastal areas and start upstream migration (Tesch, 1977; Cresci, 2020). Glass eels develop into elvers during upstream migration and settle as yellow eels in coastal and inland water habitats such as estuaries, rivers, streams, ponds and lakes (Tesch, 1977; Cresci, 2020). They start downstream migration during the silver eel stage upon gonad maturation and return to the offshore spawning area where they spawn and die (Tesch, 1977; Cresci, 2020). Otolith microchemistry studies have found that many yellow and silver eels of temperate and tropical anguillid eels stayed their entire lives in coastal waters without freshwater lives (e. g., Tzeng et al., 1997; Tsukamoto et al., 1998; Daverat et al., 2006; Jessop et al., 2008; Arai and Chino, 2012, 2018; Yokouchi et al., 2012; Marohn et al., 2013; Arai et al., 2020). Furthermore, these migratory history studies have also found diverse migration patterns between marine and freshwater habitats in anguillid eels. The estuarine residence was clearly revealed, and it became evident that some eels switched their habitats between various saline water environments. The diverse migration patterns during their growth phase suggest that a certain proportion of eels frequently shift their habitats in different saline environments. Therefore, anguillid eels do not necessarily enter freshwater habitats; thus, the eels are believed to exhibit facultative catadromy (e. g., Tzeng et al., 1997; Tsukamoto et al., 1998; Daverat et al., 2006; Jessop et al., 2008; Arai and Chino, 2012, 2018; Yokouchi et al., 2012; Marohn et al., 2013; Arai et al., 2020). However, the mechanism by which some anguillid eels immigrate into freshwater while others do not is not clear. Diadromous fish migration can typically be explained by a difference in food availability between freshwater and marine habitats (Gross, 1987). Diadromous migration results in optimal trade-offs between benefits and costs, which differ between habitat environments. Anadromous salmonids live in freshwater habitats during juvenile growth with low productivity at higher latitudes, but they migrate to the ocean for growth with higher productivity before migrating back to freshwater spawning areas. In contrast, in catadromous migration, anguillid eels recruiting in lower latitudes may immigrate into freshwater habitats with higher productivity for growth before returning to offshore spawning areas.

This paper examines the ancestral oceanic migration mechanisms observed in anguillid eels and how anguillid eels evolved to endure long-distance migratory paths to return from their temperate growth habitats to their tropical spawning areas. Furthermore, causes of upstream migration plasticity and why the migration into freshwater habitats is not an obligatory behaviour are also discussed. Recent ecological studies in combination with molecular genetic studies and the availability of electronic tags such as pop-up satellite archival tag are gradually uncovering the mysterious life history of anguillid eels.

## 2 Where did anguillid eels originate?

Currently, nineteen species and subspecies of the genus *Anguilla* have been recognized (Ege, 1939; Castle and Williamson, 1974; Watanabe et al., 2004, 2009, 2013, 2014a, b; Arai, 2016) (Figure 1). Ege (1939) first classified the genus into nineteen species and subspecies, i.e., *A. celebesensis* Kaup, 1856; *A. interioris* Whitley, 1938; *A. ancestralis* Ege (1939); *A. megastoma* Kaup, 1856; *A. nebulosa nebulosa* McClelland, 1844; *A. nebulosa labiata* Peters, 1852; *A. marmorata* Quoy and Gaimard, 1824; *A. reinhardtii* Steindachner, 1867; *A. borneensis* Popta, 1924; *A. japonica* Temminck and Sclegel, 1846; *A. rostrata* Lesueur, 1817; *A. anguilla* Linnaeus, 1758; *A. diefenbachii* Gray, 1842; *A. mossambica* Peters, 1852; *A. bicolor pacifica* Schmidt, 1928; *A. bicolor bicolor* McClelland, 1844; *A. obscura* Günther, 1872; *A. australis australis* Richardson, 1841 and *A. australis scmidtii* Phillips 1925. Thereafter, *A. ancestralis* was a synonym of *A. celebesensis* (Castle and Williamson, 1974). Watanabe et al. (2014b) further determined that *A. nebulosa nebulosa* and *A. nebulosa labiata* are synonyms of *A. bengalensis bengalensis* Gray 1831 and *A. bengalensis labiata* Peters 1852, respectively. Two new species, *Anguilla luzonensis* Watanabe et al. (2009) (Watanabe et al., 2009) and *A. huangi* Teng et al. (2009) (Teng et al., 2009) were recently found in the northern Philippines. However, Watanabe et al. (2013) suggested that *A. huangi* is a junior synonym of *A. luzonensis*. Anguillid eels are distributed nearly world-wide except for the South Atlantic and the eastern Pacific oceans (Ege, 1939; Tesch, 1977) (Figure 1).

Several evolutionary hypotheses for freshwater eels have been examined in relation to morphological (Ege, 1939) and molecular genetic (Bastrop et al., 2000; Aoyama et al., 2001; Lin et al., 2001; Minegishi et al., 2005; Tseng et al., 2009; Tseng, 2016) characteristics. Some questions have consistently bewildered us about evolution in anguillid eels. When and where did anguillid eels originate? Which species of anguillid eels is the most ancestral? The recent development of molecular research has allowed us to address some of these questions in combination with morphological characteristics and ecological implications.

**Figure 2:**
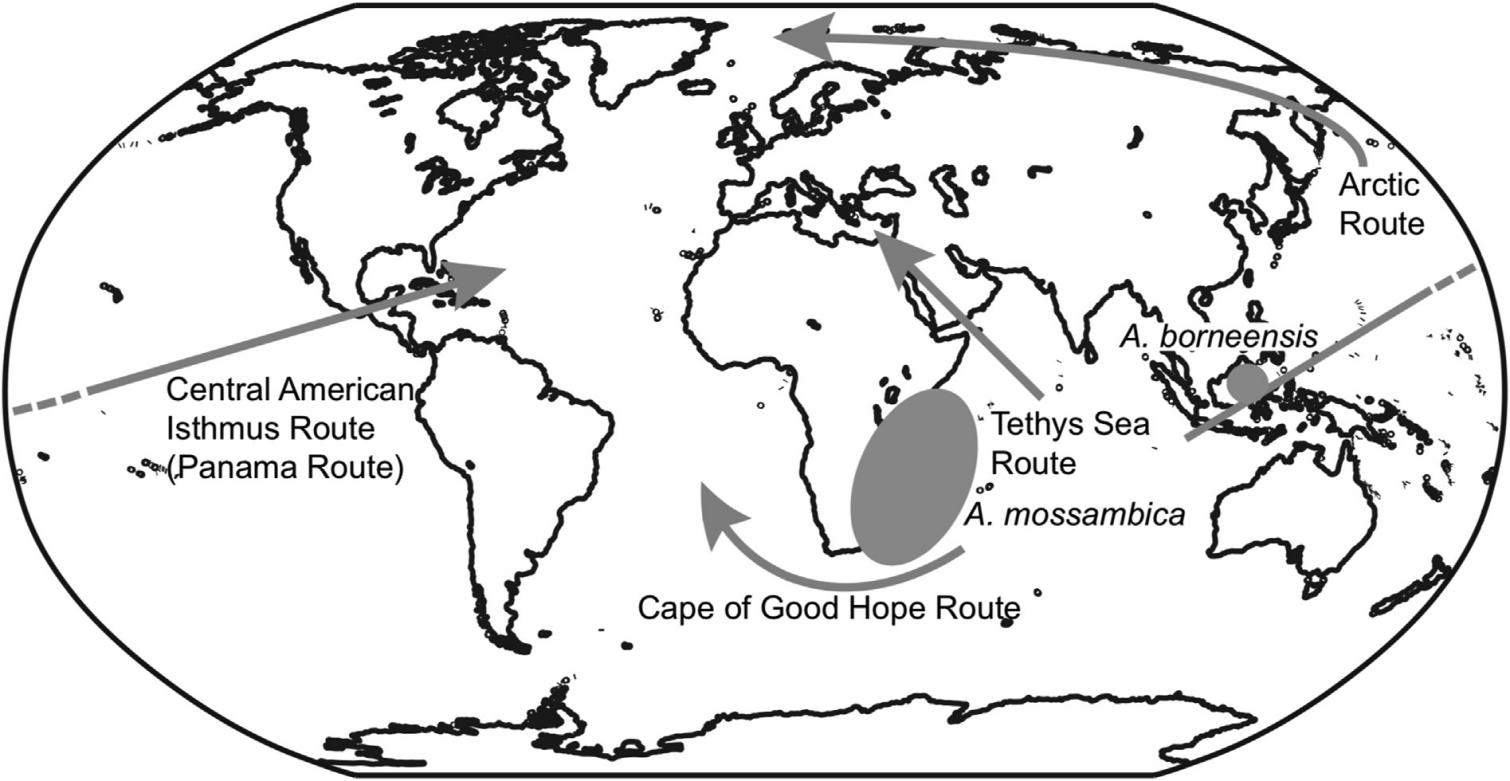
Potential dispersal routes of ancestral anguillid eels into the Atlantic Ocean and distributions of the tropical anguillid eels *Anguilla borneensis* and *A. mossambica* which are thought to be the most basal species of the anguillid eels.

Evolutionary studies present different results addressing questions about the most likely ancestral species and dispersal patterns and routes. Thus far, six possible dispersal routes have been proposed: (1) the Tethys Sea route (Bastrop et al., 2000; Aoyama et al., 2001); (2) the Cape of Good Hope route (Minegishi et al., 2005; Tseng, 2016); (3) the Central American Isthmus route (Panama route) (Lin et al., 2001; Teng et al., 2009); (4) the Arctic route (Minegishi et al., 2005; Tseng, 2016) (Figure 2); (5) multidirectional dispersion (Minegishi et al., 2005); and (6): multiple radiation events (Lin et al., 2001; Teng et al., 2009). Appropriate molecular phylogenetic studies suggest that the genus *Anguilla* originated from the tropical Indo-Pacific region and that *A. borneensis* or *A. mossambica* may be an ancestor distributed across the Indo-Pacific Ocean (Aoyama et al., 2001; Lin et al., 2001; Minegishi et al., 2005; Teng et al., 2009; Tseng, 2016; Zan et al., 2020). It is absolutely certain that the Indo-Pacific region is the original central location of speciation in anguillid eels. At present, most anguillid eels (13 species/subspecies) are distributed across tropical areas and six species/subspecies are found in temperate areas (Ege, 1939; Castle and Williamson, 1974; Watanabe et al., 2004, 2009, 2013, 2014a, b; Arai, 2016). Although eel researchers pay more attention to temperate eels, studying the biological and ecological features of tropical eels will provide functional direction and a deeper understanding of anguillid eels in nature.

Anguillid eels are thought to have occurred during the Eocene (Ypresian, approximately 50–55 Mya) based on fossil records (Patterson, 1993). The history of evolution in anguillid eels can be retraced to 50 Mya. However, based on molecular clock analysis, the estimated divergence time of anguillid eels is 52 Mya and the speciation of extant anguillid species began at approximately 20 Mya (Minegishi et al., 2005). However, a slight discrepancy is found between fossil and molecular evidence, as the molecular evidence deduces a more recent speciation sketch. The divergence time inferred from molecular evidence is thought to be underestimated due to particular ecological characteristics in relation to a slow metabolism (Minegishi et al., 2005). In contrast, the appearance of *Anguilla* is estimated to approximately 55 Mya according to fossil records and several radiation events likely appeared from 20–55 Mya (Teng et al., 2009). However, these speculations should be patently revealed with more essential evidence.

All anguilliform eels descended from a marine ancestor (Johnson et al., 2011). A molecular phylogenetic study has revealed that anguillid eels were derived from deep-ocean midwater marine anguilliform species (Inoue et al., 2010). This suggests that anguillid eels have retained their ancestral offshore spawning areas in tropical waters but that they were able to switch from a pelagic lifestyle to benthic behaviours after recruiting freshwater areas for their juvenile growth phase. As ancestral anguillid eels originated from deep-ocean areas of the Indo-Pacific Ocean, catadromous life histories might also originate from tropical regions, establishing a regular pattern of entering freshwater for growth and then returning to deep ocean areas for spawning. The migration loop extending to coastal waters that incidentally visited estuaries and eventually obtained a reproductive advantage because of higher food availability in estuaries and then in freshwater (Tsukamoto et al., 2002). Thus, the ancestor of anguillid eels probably developed an adaptive behaviour of regularly migrating upstream as a result of a cline in food abundance between the ocean and freshwater in the tropics (Gross, 1987). In their freshwater habitats, fewer competitors for food resources compared to those of marine habitats may exist, and anguillid eels may be able to extend their geographical distribution to maintain their unique catadromous migratory patterns.

## 3 Evolution of oceanic migration

Catadromous anguillid eels undergo two oceanic migratory phases throughout their lives, i.e., early-stage oceanic migration from hatching to recruitment and oceanic spawning migration. Spectacular long-distance migratory paths from eels’ continental habitats to their spawning areas in the ocean still remain a mystery of their ecology. The spawning areas of *A. anguilla* and *A. rostrata* were discovered in the Sargasso Sea of the Atlantic Ocean (Schmidt, 1922). The spawning sites are located thousands of kilometres far from their continental growth habitats in Europe and North America (Schmidt, 1922; Miller et al., 2019), demonstrating that the Atlantic eels assemble prominently large-scale spawning migrations. The European eel spawns across at least a 2000-km area of the Sargasso Sea (Miller et al., 2019). The reasons for such a wide spawning area may be related to how natural selection has interacted with both the physiological constraints on the approximately 5000–7000-km long adult migration and subsequent reproductive success (Miller et al., 2019). The spawning site of *A. japonica* is located in the western North Pacific Ocean (Tsukamoto, 1992; Tsukamoto et al., 2011). Notably, all developmental stages in the ocean, such as fully matured adults, eggs, and newly hatched larvae, have also firstly been collected in *A. japonica* among 19 species/subspecies in the genus *Anguilla* (Tsukamoto et al., 2011). The spawning sites in the Atlantic and Pacific Oceans are commonly located in westward flowing currents of the southern edges of the subtropical gyres. Hence, those leptocephali can be passively drifted to continental habitats. The spawning areas of the Australasian temperate anguillid eels, *A. australis* and *A. dieffenbachii*, in the western South Pacific Ocean are assumed to be located in the South Equatorial Current based on larval collections (Jellyman, 1987, 2003) and migratory behaviour and routes of the silver eels in the ocean (Jellyman and Tsukamoto, 2010). The spectacular thousands of kilometres of large-scale migrations in temperate species have attracted scientists, as all eels must return to the same site for breeding. Discoveries of the spawning sites in temperate species have enhanced a number of ecological and biological studies on their life histories.

Compared to temperate anguillid species, there is very little known about the spawning sites of tropical anguillids. The spawning areas of *A. marmorata* and *A. mossambica* in the southwestern Indian Ocean were suggested to be localized northeast off the Mascarene Ridge (Jespersen, 1942; Jubb, 1961; Robinet et al. 2003a, 2008; Réveillac et al., 2008, 2009), and it has been further postulated that the area could lie between Madagascar Island and the Mascarene Archipelago (Robinet et al., 2008; Réveillac et al., 2008, 2009). A spawning site of *A. bicolor bicolor* was proposed around the Mentawai Trench, off Sumatra Island of western Indonesia (Jespersen, 1942). Spawning areas of *A. celebesensis* and *A. borneensis* were assumed to locate in the Celebes Sea and in the Tomini Bay and Celebes Sea, respectively, off north and central Sulawesi Island of eastern Indonesia (Aoyama et al., 2003, 2018; Arai, 2014a). Small larvae of *A. celebesensis* and *A. borneensis* were found in the Celebes Sea (Jespersen, 1942; Aoyama et al., 2003, 2018), close to their continental habitats in North Sulawesi and East Borneo (Kalimantan). These two species can spawn sympatrically in the same site, and that the migration scale of *A. borneensis* should result in a small scale, such as *A. celebesensis*. Interestingly, the spawning sites of *A. celebesensis* have been discovered in at least two areas in the Celebes Sea and Tomini Bay around Sulawesi Island (Aoyama et al., 2003, 2018; Arai, 2014a). Aoyama et al. (2018) found that the spawning site of *A. interioris* is located in Tomini Bay and suggested the species has other spawning areas in the Indonesian Seas. The multiple spawning sites in *A. celebesensis* and *A. interioris* were definitely in contrast to temperate species. It is assumed that western South Pacific eels such as *A. marmorata*, *A. megastoma*, *A. obscura* and *A. reinhardtii* spawn somewhere within the South Equatorial Current (Jellyman, 2003; Schabetsberger et al., 2013, 2015; 2019; Hewavitharane et al., 2020), and it has been suggested these western South Pacific eels may migrate a short distance between their continental habitats and spawning sites by means of recently developed pop-up satellite archival tag (PSAT) studies (Schabetsberger et al., 2013, 2015, 2019). These findings suggest that certain tropical anguillids exhibit considerably short-scale migrations between spawning areas and continental habitats compared to temperate species that make large-scale migrations.

Migrating silver eels of the Atlantic and Japanese eels have been fortuitously collected through continental margins (Wenner, 1973; Ernst, 1977; Bast and Klinkhardt, 1988; Kotake et al., 2005; Chino and Arai, 2009) but hardly caught in those spawning sites except for the Japanese eel (Tsukamoto et al., 2011). Female migrating European silver eels usually have gonadosomatic index (GSI) values of more than 1.2 (Vøllestad and Jonsson, 1986; Durif et al., 2005) but not more than 3.0 (Svedäng and Wickström, 1997; Durif et al., 2005). The GSI values of Japanese eels have been reported to range from 1.0 to 4.0 in Japanese coastal areas (Kotake et al., 2007). In contrast, the GSI values of *A. celebesensis* silver eels in an inland freshwater lake were more than 4.0 (Hagihara et al., 2012; Arai, 2014a). Considerably high GSI values of more than 9.0 are found in *A. celebesensis* from the uppermost lake (Lake Poso) in central Sulawesi of Indonesia (Hagihara et al., 2012; Arai, 2014a). The lake eventually drains to Tomini Bay via a 40-km-long stretch of the Poso River. Such a remarkably short-distance migration for spawning to Tomini Bay close to the river mouth of the Poso River can stimulate the final maturation preparations in the lake in a short time to reach the spawning site (Arai, 2014a). Although the oogenesis of *A. celebesensis* is remarkably advanced at the timing of initiation of downstream migration (Arai, 2014a; Hagihara et al., 2020), they do not spawn immediately and require more time for spawning (Hagihara et al., 2020). *A. celebesensis* is thought to need a few months for spawning after initiation of their downstream migration based on the oocyte developmental stage and the downstream migration season, although the timing of downstream migration was not examined monthly throughout the year (Hagihara et al., 2012, Hagihara et al., 2020). A maturing *A. rostrata* was completely tracked for 2400 km during its spawning migration between the Canadian east coast (Scotian Shelf) and the northern limit of the spawning site in the Sargasso Sea by means of PSATs. It reached the spawning site 45 days after release (Béguer-Pon et al., 2015). Similarly, in the case of *A. celebesensis* from the mouth of Lake Poso, eels would reach the spawning site in the Tomini Bay in a few days. Therefore, the mid-vitellogenic oocytes of the gonads suggest that *A. celebesensis* silver eels might ready to spawn at any time, although no study has evaluated spawning timing or how long it takes after reaching the spawning site.

A clear difference in GSI values in tropical eels between *A. celebesensis* and *A. marmorata* silver eels was found in Lake Poso of central Sulawesi of Indonesia (Hagihara et al., 2012; Arai, 2014a). Their maturation processes would be related to the distance between growth habitats and spawning areas. Migrating *A. celebesensis* had GSI values that were approximately two times higher than those of migrating *A. marmorata* (Hagihara et al., 2012; Arai, 2014a). The difference in the maturation level at the onset of spawning migration between *A. japonica* and *A. anguilla* is suggested to be reflected by the differences in their migration distances between growth habitats and spawning areas (3000 km in *A. japonica*, 6000 km in *A. anguilla*) (Yokouchi et al., 2009). Robinet et al. (2003b) also suggested a possible relationship between advanced sexual maturation of *A. bicolor bicolor* from Réunion Island (6.78 in GSI) and the assumed spawning area (between 10–20 °S and 60–65 °E). Differences in GSI values of *A. dieffenbachii* and *A. australis* at the beginning of their spawning migration were also found, a GSI of 8.1 was recorded in the former species and a GSI of 3.5 in the latter species (Todd, 1981). This difference in the maturation level suggests that spawning migration of *A. dieffenbachii* would be shorter than that of *A. australis* (Jellyman, 1987). Although *A. dieffenbachii* did not show evidence of a short distance migration and probably spawn in the south Fiji basin by means of a pop-up tag study (Jellyman and Tsukamoto, 2010), it may still be a shorter spawning migration than that of *A. australis*, which appears to spawn farther north in the South Equatorial Current (Jellyman, 2003; Schabetsberger et al., 2013).

*A. marmorata* in Sulawesi Island belongs to the North Pacific Ocean population (Ishikawa et al., 2004; Minegishi et al., 2008), and the spawning area was discovered in the North Equatorial Current region of the western North Pacific Ocean, which overlaps with that of the Japanese eel *A. japonica* (Tsukamoto et al., 2011). Aoyama et al. (2018) also suggested that *A. marmorata* migrates out of the Indonesian Seas to spawn in the North Pacific based on the larger larvae size distributions in the seas. *A. japonica* and *A. marmorata* are suggested to share and have overlapping larval transportation routes and durations, although the geographical distributions in these eels are allopatric in the western Pacific region (Han et al., 2012b). The spawning migration of *A. marmorata* ranges from 1000 km to 3000 km and is similar to that of *A. japonica* (Han et al., 2012b; Arai, 2014a). The differences in the preferences of the recruitment temperature for glass eels and the availability of oceanic currents shape the real geographic distribution of *A. japonica* and *A. marmorata*, making them ‘‘temperate’’ and ‘‘tropical’’ eels, respectively (Han et al., 2012b). A similar migration scale in *A. marmorata* was found in the southwestern Indian Ocean (Jespersen, 1942; Jubb, 1961; Robinet et al., 2008; Réveillac et al., 2008; Gagnaire et al., 2009). The spawning area of *A. marmorata* was suggested to be located 1000–1500 km off the east coast of Madagascar (Robinet et al., 2008; Réveillac et al., 2008; Gagnaire et al., 2009). Interestingly, the migration scale of *A. marmorata* was much longer than those of *A. celebesensis* and *A. borneensis*. *A. marmorata* has the widest geographic distribution from temperate to tropical areas among all anguillid eels (Ege, 1939) and is found longitudinally from the east coast of Africa to the Marquesas Islands in the southeast Pacific Ocean and as far north as southern Japan (Ege, 1939). This species was also discovered in the central Pacific (Handler and James, 2006) and in the Galapagos Islands (McCosker et al., 2003). These findings suggest that the geographic range of *A. marmorata* would be wider than previously thought. The larger larval dispersal in the ocean and the higher adaptation for the ambient environment in continental growth habitats from tropical to temperate areas may presumably result in the longer distance spawning migration and wider distribution range in *A. marmorata* compared to other tropical eels such as *A. celebesensis* and *A. borneensis*; thus, it can be categorized as a mid-scale migratory species. The population genetic structure of *A. marmorata* revealed that this species has four populations (North Pacific, South Pacific, Indian Ocean, and Guam region) (Minegishi et al., 2008). However, the exact spawning site has been discovered for only one of the North Pacific populations (Tsukamoto et al., 2011) among the four populations in *A. marmorata*.

Temperate anguillid species undergo downstream migration to offshore spawning areas in the fall and winter (Todd, 1981; Sloane, 1984; Vøllestad and Jonsson, 1986; Jellyman, 1987; McCleave et al., 1987; Kotake et al., 2007; Aarestrup et al., 2008; Acou et al., 2008; Tsukamoto et al., 2011; Han et al., 2012a, 2016b; Verreault et al., 2012; Reckordt et al., 2014; Sandlund et al., 2017). There has been a lack of evidence regarding downstream migration in tropical anguillid eels due to the difficulties involved in catching migrating silver eels in coastal waters. Recently, however, spawning of *A. bicolor bicolor* and *A. bengalensis bengalensis* females was found to occur throughout the year (Arai et al., 2016; Arai and Abdul Kadir, 2017a). The continuous spawning is suggested to be a consistent spawning ecology of tropical anguillid eels. The first discovery of the year-round spawning indicates that the life history of tropical eels considerably differs from that of temperate counterparts. However, studies on the downstream migration and spawning ecology of tropical eels species are relatively less than those of temperate eel species; and thus, further research is needed to emphasize the unique characteristics in tropical anguillid species.

**Figure 3:**
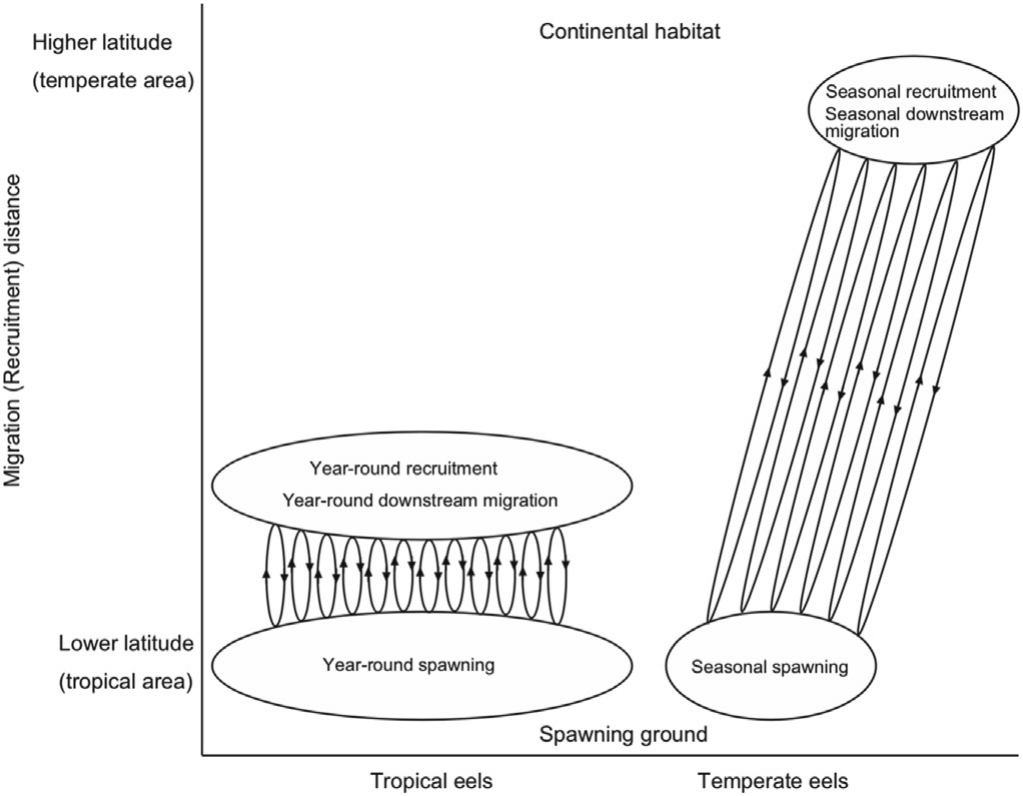
Scenario illustrating the evolution of anguillid eel migration from local short-distance movements with year-round spawning, recruitment and downstream migration for tropical eels to long-distance migration with seasonal spawning, recruitment and downstream migration for temperate eels.

The spawning sites of anguillid eels are all located in southern areas near the equator (Schmidt, 1922; Tsukamoto, 1992; Tsukamoto et al., 2011; Miller et al., 2019). The early life histories such as the ages at recruitment to coastal habitats in tropical eels were constant throughout the year (Arai et al., 2001). The spawning occurred through the whole year in several tropical eel species (Arai et al., 2001; Shiao et al., 2002; Arai et al., 2016; Arai and Abdul Kadir, 2017a). The year-round spawning together with constant early growth during the oceanic period throughout the year, expand the recruitment period to continental habitats throughout the entire year for tropical anguillid eels (Beumer and Sloane, 1990; Arai et al., 1999b; Sugeha et al., 2001; Han et al., 2012b, 2016a; Leander et al., 2012; Aoyama et al., 2015; Hewavitharane et al., 2018). The continuous spawning migration may stimulate breeding with different cohorts (year classes) or divergent habitats within the population. Local short-distance migrations facilitate such spawning migration and inshore migration mechanisms (Figure 3).

Seasonal spawning was suggested in certain tropical eels based on the larval distribution in Indonesian Seas (Aoyama et al., 2003, 2018; Wouthuyzen et al., 2009). However, it would be difficult to estimate the spawning period using a few survey data of larval distribution in the open ocean and hatching date estimations using those larval otoliths. Compared to temperate eels, such as Atlantic and Japanese eels, larval collections and oceanographic research are highly limited, and there are quite sporadic and limited data available for all tropical eel species. In the European eel, historical larval collections made from 1921 to 2007 revealed that small larvae of the European eel had been spawning across a 2000-km-wide region of the North Atlantic Ocean (Miller et al., 2019) approximately 100 years after the first discovery of the spawning site in the species (Schmidt, 1922). For the Japanese eel, intensive and continuous surveys have been conducted for more than 20 years after the first discovery of the spawning ground (Tsukamoto, 1992; Tsukamoto et al., 2011; Arai, 2014c). Therefore, intensive observation of the timing of downstream migration in continental habitats and hatching date estimation by means of otolith analyses using glass eels recruited to coastal areas would be a more realistic and accurate way to know the spawning season of anguillid eels.

Ancestral eels feasibly underwent a catadromous migratory transition from localized short-range paths through tropical coastal waters by means of simple migratory mechanisms, consequently evolving the large-scale migratory paths of currently temperate eels, which are well developed in subtropical gyres (Figure 3). The long larval duration from approximately 3 to 24 months in anguillid eels (e.g. Tsukamoto, 1990; Lecomte-Finiger, 1992; Cheng and Tzeng, 1996; Arai et al., 1999a, 2000; 2001, 2002; 2003a; Wang and Tzeng, 2000; Marui et al., 2001; Robinet et al., 2003a, 2008; Réveillac et al., 2008, 2009; Bonhommeau et al., 2010; Han et al., 2016a, 2019; Hewavitharane et al., 2020) seems to highly adjust marine planktonic life. Passive transportation by oceanic currents and winds during the slow leptocephalus growth would facilitate the global dispersion almost across the world. The accidental drifting of larvae through global circum-equatorial currents and early life variations could expand their geographical distribution. Temperate anguillid eels would need to migrate thousands kilometers of long distance because the eels retain spawning sites in tropical waters at low latitude (Figure 3).

## 4 Plasticity of continental migration

Anguillid eels settle down in waters at a wide range of salinities, from saline coastal waters and estuaries to freshwater rivers after recruitment to coastal areas (Tesch, 1977; Marohn et al., 2013). Their habitat environments cause differences in their growth rate or sex ratio (Morrison and Secor, 2003; Davey and Jellyman, 2005; Yokouchi et al., 2008; Côté et al., 2009); hence, their habitats play an important role in their life history traits through their plastic response to environmental conditions. Marohn et al. (2013) suggested that coastal areas are important growth habitats in *A. anguilla*. The Baltic Sea represents a very special habitat for *A. anguilla* because the brackish water system resembles an enlarged estuarine habitat (Marohn et al., 2013). Less fluctuating salinity and predators and rich prey resources for eels in the Baltic Sea appeared as a result of facultative catadromous behavior, with fewer migration strategies in their habitats (Marohn et al., 2013). It was also suggested that the genetically (and/or epigenetically) based differences caused by spatially varying selection could be related to regional differences in eel life history traits (Côté. et al., 2009, 2014; 2015; Yokouchi et al., 2012; Gaillard et al., 2016). Yokouchi et al. (2012) suggested that well-suited freshwater habitats would be occupied by early recruitment glass eels and that movement is less beneficial for eels because movement from a certain growth habitat would be a risk to find a better habitat. There are not only continuous freshwater or marine residents but also habitat shifters that have multiple movements between saline water and freshwater during the continental growth period (Tsukamoto and Arai, 2001; Jessop et al., 2002; Daverat and Tomás, 2006; Yokouchi et al., 2012). These shifters would not be able to find suitable habitats during their growth phase; hence, they would move habitats frequently.

Diverse migratory histories in all temperate eels and several tropical eel species have been found by means of otolith microchemical analyses determining concentration ratios of strontium (Sr) to calcium (Ca) (e.g., Tzeng et al., 1997; Tsukamoto et al., 1998; Daverat et al., 2006; Jessop et al., 2008; Arai and Chino, 2012, 2018; Yokouchi et al., 2012; Marohn et al., 2013; Arai et al., 2020). Sr concentration and Sr:Ca rations in eel otoliths have been found to be positively increased with the salinity, while they are less affected by other factors, such as water temperatures, food and physiological factors (Tzeng, 1996; Kawakami et al., 1998; Lin et al., 2007; Arai and Chino, 2017). Therefore, otolith Sr:Ca ratios could reconstruct the migratory history of whether each eel truly immigrates and settles into freshwater, estuarine or marine environments until they reach the silver eel stage or whether they move between different saline waters. Otolith Sr:Ca ratios revealed that many eels stayed their whole lives in marine waters without freshwater lives after recruitment to coastal waters in the temperate eels *A. anguilla* (Tzeng et al. 1997, 2000; Tsukamoto et al., 1998; Arai et al., 2006, 2019; Daverat et al., 2006; Marohn et al., 2013), *A. rostrata* (Jessop et al., 2002, 2008), *A. japonica* (Tsukamoto and Arai, 2001; Arai et al., 2003b, c, 2008; Shiao et al., 2003; Yokouchi et al., 2012) and *A. dieffenbachii* and *A. australis* (Arai et al., 2004) and the tropical eels *A. marmorata* (Shiao et al., 2003; Briones et al., 2007; Chino and Arai, 2010 a; Lin et al., 2012; Arai et al., 2013; Arai and Chino, 2018), *A. mossambica* (Lin et al., 2012), *A. bicolor bicolor* (Chino and Arai, 2010b, c; Arai and Chino, 2019; Arai et al., 2020) and *A. bicolor pacifica* (Briones et al., 2007; Arai et al., 2013; Arai and Chino, 2019). Furthermore, alternative migratory histories of the estuarine residents were also found in these studies, indicating that eels frequently shifted their habitats between marine and freshwater environments in different salinity regimes. The migratory history studies revealed that all eels do not necessary use freshwater environments; thus, anguillid eels exhibit a facultative catadromy.

At present, it is unknown why, during continental migration, some eels immigrate and settle into freshwater habitats while others do not migrate. In general, diadromous fish migration is spurred by a difference in food resources between freshwater and marine habitats (Gross, 1987). Based on this theory, catadromous anguillid eels recruiting in lower latitudes are assumed to migrate to freshwater habitats and settle there until starting the downstream migration to offshore spawning sites, as freshwater environments have higher productivity than marine environments. A latitudinal cline in habitat uses and migration patterns in anguillid eels may be predicted because the primary production in freshwater habitats is lower than that of coastal and marine habitats at higher latitudes; hence, marine and estuarine resident eels will occur more frequently (Tsukamoto et al., 2002). However, wide ranges of otolith Sr:Ca ratios were found in tropical eels such as *A. marmorata, A. mossambica*, *A. bicolor bicolor* and *A. bicolor pacifica* (Shiao et al., 2003; Briones et al., 2007; Chino and Arai, 2010 a, b, c; Lin et al., 2012; Arai et al., 2013, 2020; Arai and Chino, 2018, 2019), suggesting that tropical eel species show facultative migratory histories. The habitat use and choice of tropical eels, whether or not individual eels actually enter freshwater and remain in freshwater, estuarine or marine environments until the silver-eel stage or whether they move between different habitats with differing salinity regimes, are the same as those found in temperate anguillid eel species (Tzeng et al., 1997; Tsukamoto et al., 1998; Tzeng et al., 2000; Tsukamoto and Arai, 2001; Jessop et al., 2002, 2008; Shiao et al., 2003; Arai et al., 2003b, c, 2004, 2006, 2008, 2019; Daverat et al., 2006; Arai and Chino, 2012; Yokouchi et al., 2012; Marohn et al., 2013). Therefore, the latest results on habitat use among tropical eels do not support latitudinal clines of migratory plasticity for anguillid eels. To reside in various salinities during the continental growth phase could be a common behaviour in all anguillid species without latitudinal clines. The results suggest that habitat use and migratory pattern would be influenced by environmental situations and/or by levels of intra- and/or inter-species competition found in each site. Recently, Fukuda et al. (2019) found that shifts in salinity preference occurred from the glass eel stage in winter to the yellow eel stage in fall in *A. japonica*. This shift in salinity preference would be the reason for the occurrence of estuary residents (Fukuda et al., 2019). When eels grow in saline water until the yellow eel stage, they tend to be estuary residents (Fukuda et al., 2019).

Anguillid eels are believed to derive from a marine ancestor, and all anguilliform fishes besides anguillds are marine species (Inoue et al., 2010); hence, the oceanic spawning habits would be a conservative feature. Thus, many anguillid eels live in coastal and marine habitats after recruitment, while it is not clear whether the occurrence of marine and estuarine resident eels is due to a remnant genetic feature or ecological plasticity to utilize the full range of habits. It has been found that eel groups of different migration types exhibit no apparent genetic differentiation in *A. japonica* (Han et al., 2008, 2010). Therefore, variations in habitat use are more likely to be a result of behavioural plasticity, which depends on external factors such as habitat environments and/or intra- and/or interspecific competition.

In temperate regions, there are no plural anguillid eel species distributed in the continental habitats of each region except in New Zealand where *Anguilla dieffenbachii* and *A. australis schmidtii* are distributed sympatrically (Glova, 1988; Glova et al., 1998). However, several species are distributed sympatrically in tropical areas and the habitat preferences also rely on the occurrence of multiple species within a habitat or a single species (Robinet et al., 2007; Arai and Chino, 2012, 2018; Arai et al., 2013; Arai and Abdul Kadir, 2017b; Hagihara et al., 2018; Nguyen et al., 2018; Hsu et al., 2019; Arai et al., 2020). In rivers in the Réunion and Mauritius islands of the western Indian Ocean, *A. marmorata* showed a strong altitudinal distribution from the lower to upper areas, while *A. mossambica* was only found in the upper areas and *A. bicolor bicolor* occurred only in the lower areas (Robinet et al., 2007). *A. marmorata* was mainly distributed in upstream freshwater environments in Taiwan and the Philippines (Shiao et al., 2003; Briones et al., 2007; Hsu et al., 2019), while the species show diverse migratory patterns in Indonesia, Japan and Vietnam (Arai and Chino, 2018). In the Philippines and Taiwan, *A. bicolor pacifica* and *A. marmorata* and *A. marmorata* and *A. japonica*, respectively, are distributed sympatrically (Shiao et al., 2003; Briones et al., 2007; Hsu et al., 2019), while there are only *A. marmorata* distributed in the Amami and Bonin Islands of Japan (Arai and Chino, 2018). *A. marmorata* can settle in various environments from river upstream to downstream in those islands in Japan without interspecific competition; thus, estuarine resident eels would be more plentiful, with no habitat segregation between species there compared to Taiwan and the Philippines. There is habitat segregation between *A. australis* and *A. dieffenbachii* in New Zealand. *A. australis* generally tends to live in downstream, and *A. dieffenbachii* typically settles more upstream sites (McDowall, 1990). *A. bicolor bicolor* is generally found throughout rivers, while *A. bengalensis bengalensis* is found from upstream to midstream sites in Malaysia (Arai and Abdul Kadir, 2017b; Arai et al., 2020). Such sympatric distribution of *A. bicolor pacifica* and *A. marmorata* along rivers was also found in Vietnam (Arai et al., 2013; Nguyen et al., 2018). Approximately 90% of eels showed estuarine resident migratory patterns, and the remaining eels were marine residents, while no freshwater residents were found in *A. bicolor pacifica* (Arai et al., 2013). In *A. marmorata*, approximately 90 % of eels were also estuarine residents, and the remnant eels were freshwater residents, while there were no marine residents (Arai et al., 2013). In Vietnam, *A. bicolor pacifica* prefers to reside in higher saline waters than *A. marmorata*. These results suggest that all anguillid eels might have an ability to live in a wide range of salinities during the continental growth phase, while the actual habitat use might depend on whether there are sympatric species distributed in a habitat.

Interspecific differences in the riverine distribution among sympatric species have also been found in various tropical areas, and it was suggested that environmental factors and interactive habitat segregation would affect the riverine distribution of sympatric anguillids (Cumaranatunga et al., 1997; Robinet et al., 2007; Arai and Abdul Kadir, 2017b; Hagihara et al., 2018; Arai et al., 2020). In Taiwan, *A. japonica* mainly inhabited the lower and middle reaches of rivers, while *A. marmorata* was distributed over the middle to upper reaches (Shiao et al., 2003; Hsu et al., 2019). Hsu et al. (2019) recently found that the mRNA expression levels of five candidate genes related to upstream migration were higher in *A. marmorata* than those in *A. japonica*, indicating that *A. marmorata* might have better swimming bursts and more active upstream migration than *A. japonica*. The results suggest that habitat segregation between them in the river system may be associated with active swimming and upstream migration capacities (Hsu et al., 2019).

Other potential clarifications for differences in habitat use and migratory history and behaviour observed would be rooted in variations in habitat environments observed among regions (e.g., carrying capacities, current velocities, bottom materials and inclination pitches). Habitat segregation is indicated due to physical features in habitat environments in New Zealand (Glova et al., 1998). Faster water velocities and larger substrates of riffles were preferred in *A. dieffenbachii*, while *A. australis* tended to live in slower marginal habitats (Glova et al., 1998). In Malaysia, habitat preferences vary between habitats following inter-species interactions and intraspecific plasticity to each environmental condition between *A. bicolor bicolor* and *A. bengalensis bengalensis* (Arai and Abdul Kadir, 2017b; Arai et al., 2020). The results suggest that environmental parameters such as temperature, salinity, river size, elevation and carrying capacity and ecological factors such as interspecific competition would predominantly affect habitat uses in anguillid eels. Otherwise, differences in swimming and migration behaviours and activities between species might lead to avoiding/reducing interspecific competition in the river system (Hsu et al., 2019). The migratory behaviours of anguillid eels observed during continental migration exhibit phenotypic plasticity in each habitat in accordance with variations in physical conditions and interactive habitat segregation among sympatric anguillid species in the growth habitats. Further research on the riverine distribution and habitat use of tropical eels using quantitative and environmental data in combination with genetic, physiological and morphological studies is needed to elucidate the valid mechanisms of habitat use, segregation and choice during the continental growth phase in anguillid eels.

## 5 Conclusions

Anguillid eels, genus *Anguilla*, are mysterious animals. Already in approximately 350 BC, Aristotle wrote in his *Historia Animalium* (*History of Animals*) that “the eels come from what we call the entrails of the earth” (Bertin, 1956). Science has long been searching to discover and understand the mysteries of spawning and migration in the genus *Anguilla*. It took another 2000 years for the Danish biologist Johannes Schmidt to first discover that the spawning grounds of Atlantic eels, *A. anguilla* and *A. rostrata*, are located in the Sargasso Sea according to approximately 20-year surveys (Schmidt, 1922, 1925). Although a large body of scientific literature has been published on anguillid eels, crucial aspects of their biology, mainly regarding their migration and reproduction, remain a mystery. Recent declines in temperate anguillid eel populations such as those of *A. anguilla*, *A. rostrata* and *A. japonica* (Dekker, 2003; Arai, 2014b; Jacoby et al., 2015), indicate the need to focus on their migration mechanisms, as this will support for the management, conservation and recovery of these eel populations.

A variety of recent advancements made by means of molecular genetic and ecological studies have better elucidated the ecology and evolution of anguillid eels. Scales of spawning migration distinctly differ among tropical eel species (Aoyama et al., 2003, 2018; Robinet et al., 2008; Réveillac et al., 2008, 2009; Schabetsberger et al., 2013, 2015; 2019; Arai, 2014a; Arai and Abdul Kadir, 2017a; Han et al., 2019; Hewavitharane et al., 2020). Less than 100 km of small-scale migration and 1000–3000 km of mid-scale migration are suggested in *A. clelebesensis* and *A. borneensis* (Aoyama et al., 2003, 2018; Arai, 2014a) and *A. marmorata*, *A. bicolor bicolor*, *A. mossambica* and *A. bengalensis bengalensis* (Robinet et al., 2008; Réveillac et al., 2008, 2009; Arai, 2014a; Arai and Abdul Kadir, 2017a; Aoyama et al., 2018; Han et al., 2019), respectively. Compared to their migration scales, however, temperate anguillids, such as *A. rostrata* (1000–5000 km; mid-to large-scale migration) and *A. anguilla* (5000–8000 km; large-scale migration), exhibit larger migration scales (Aoyama et al., 2003; Miller et al., 2019). The ancestral form of anguillid eel migration may have been local, small-scale migration combined with year-round spawning and recruitment, as characterized in tropical anguillid eels (Figure 3). Spawning migration scales would change gradually, accompanying the dispersion across the world. Spawning sites are located in tropical waters for all anguillids, including temperate species; thus, temperate eels need to migrate thousands kilometres, matching seasonal downstream migration and offshore spawning with subsequently seasonal recruitment to continental habitats (Figure 3).

Classical means of delimiting spawning grounds have necessitated the collection of progressively smaller leptocephali. Recent advances and the availability of electronic tags such as PSATs, data storage tags and accelerometers, marine remote sensing tools and computer modelling systems provide new means to understand the spawning migration and behaviour of anguillid eels without involving the use of expensive ocean research vessels. The development of PSATs has enabled data to record for longer periods during the oceanic migration of maturing eels to spawning sites (Aarestrup et al., 2009; Manabe et al., 2011; Schabetsberger et al., 2013, 2015; 2019; Westerberg et al., 2014; Béguer-Pon et al., 2015; Wysujack et al., 2015; Amilhat et al., 2016; Righton et al., 2016; Chen et al., 2018; Higuchi et al., 2018). As a distinct behaviour of migrating silver eels as revealed by PSATs, all eel species examined showed similar diel vertical migration (DVM) patterns, with migrating eels preferring shallower water (100–300 m) at night and deeper water (500–700 m) during the daytime (Aarestrup et al., 2009; Manabe et al., 2011; Schabetsberger et al., 2013, 2015; 2019; Westerberg et al., 2014; Béguer-Pon et al., 2015; Wysujack et al., 2015; Amilhat et al., 2016; Righton et al., 2016; Chen et al., 2018; Higuchi et al., 2018). DVMs have been observed in the temperate eels *A. dieffenbachii* (Jellyman and Tsukamoto, 2005, 2010), *A. anguilla* (Aarestrup et al., 2009; Westerberg et al., 2014; Wysujack et al., 2015; Amilhat et al., 2016; Righton et al., 2016), *A. japonica* (Manabe et al., 2011; Chen et al., 2018; Higuchi et al., 2018) and *A. rostrata* (Béguer-Pon et al., 2015) and the tropical eels *A. marmorata* (Schabetsberger et al., 2013, 2015, 2019; Chen et al., 2018), *A. obscura*, and *A. megastoma* (Schabetsberger et al., 2013, 2015, 2019) and *A. bicolor pacifica* (Chen et al., 2018).

Atlantic eels were tracked more than 2000 km along a route into the Atlantic Ocean from the Irish west coast (Aarestrup et al., 2009), the Swedish west coast (Westerberg et al., 2014) and the French south coast (Amilhat et al., 2016) in *A. anguilla* and from the east coast of Canada (Béguer-Pon et al., 2015) in *A. rostrata*. Westerberg et al. (2014) revealed the empirical evidence of the spawning migration route to the Sargasso Sea and the orientation and behaviour in *A. anguilla*. Béguer-Pon et al. (2015) firstly tracked the entire migration route from the Canadian coast to the northern limit of the spawning area in the Sargasso Sea and reconstructed daily locations of migrating eels. A temperate eel, *A. japonica*, and two tropical eels, *A. marmorata* and *A. bicolor pacifica*, showed similar migratory orientations along the Kuroshio Current from the northeast coast of Taiwan, probably because the spawning areas of *A. marmorata* and *A. bicolor pacifica* overlap with those of the Japanese eel or are nearby (Chen et al., 2018). These findings provide novel and detailed evidence of spawning migration observed among anguillid eels, such as migratory behaviours and migration routes in the ocean, for which limited information exists. However, the orientation mechanisms are still not clear: for example, it is not known if the eels take a straight compass route towards the spawning area or if they take advantage of ocean currents to gain speed and save energy. Migrating eels have a magnetic compass that they can use for orientation in *A. anguilla* (Durif et al., 2013), and *A. anguilla* glass eels also use their magnetic compass to memorize the magnetic direction of tidal flows, helping them to maintain their position in an estuary and to migrate upstream (Cresci et al., 2019). Migration mechanisms, including orientations, behaviours and routes throughout the entire lives of anguillid eels, are gradually revealed by means of the recent advanced technologies (Aarestrup et al., 2009; Manabe et al., 2011; Schabetsberger et al., 2013, 2015; 2019; Westerberg et al., 2014; Béguer-Pon et al., 2015; Wysujack et al., 2015; Amilhat et al., 2016; Righton et al., 2016; Chen et al., 2018; Higuchi et al., 2018). Interestingly, stocked and farmed eels showed similar migratory behaviour and routes during spawning migrations in open ocean (Westerberg et al., 2014; Chen et al., 2018), and these findings provide important evidence for the eel restocking programme to sustain and recovery natural eel resources. Certain tropical eels such as *A. borneensis* and *A. celebesensis* exhibit a small-scale migration between the spawning area and continental habitats; which would have a realistic and higher chance of revealing the spawning migration and behaviour. The research for these tropical eels together with further intensive research for temperate eels during oceanic migration for spawning are needed to understand their comprehensive migration routes and orientation mechanisms.

Further ecological and molecular genetic studies in combination with advanced electronic tag technologies should reveal additional mysterious ecological features of anguillid eel migration throughout their entire life since their first discovery more than 2000 years ago, with consequences for eel stock conservation and management.

## Declarations

### Author contribution statement

All authors listed have significantly contributed to the development and the writing of this article.

### Funding statement

This work was supported in part by 10.13039/100009100Universiti Brunei Darussalam under the Faculty/Institute/Centre Research Grant (No. UBD/RSCH/1.4/FICBF(b)/2019/021, UBD/RSCH/1.4/FICBF(b)/2020/029) (Universiti Brunei Darussalam, Brunei Darussalam).

### Competing interest statement

The authors declare no conflict of interest.

### Additional information

No additional information is available for this paper.
